# Enhancing Recruitment Using Teleconference and Commitment Contract (ERUTECC): a stepped wedge cluster randomised trial within the EFFECTS trial

**DOI:** 10.48101/ujms.v130.12897

**Published:** 2025-12-01

**Authors:** Eva Isaksson, Per Näsman, Per Wester, Ann Charlotte Laska, Erik Lundström

**Affiliations:** a Karolinska Institutet, Department of Clinical Sciences, Danderyd Hospital, Stockholm, Sweden; b Center for Safety Research, KTH Royal Institute of Technology, Stockholm, Sweden; c Department of Public Health & Clinical Medicine, Umeå University, Umeå, Sweden; d Department of Medical Sciences, Neurology, Uppsala University, Uppsala, Sweden

**Keywords:** Stroke, randomised controlled trials, recruitment, randomised stepped-wedge cluster trial

## Abstract

**Background:**

Two out of three randomised controlled trials (RCTs) fail to meet their recruitment goals. Recruitment to Efficacy oF Fluoxetine – a randomisEd Controlled Trial in Stroke (EFFECTS), fluoxetine for stroke recovery was slower than anticipated. We aimed to evaluate an intervention to improve recruitment to EFFECTS.

**Methods:**

This stepped wedge, cluster randomised study investigated whether a teleconference with the study personnel and the head of department could enhance recruitment in the ongoing EFFECTS. We included 20 low- and medium recruiting active centres. We excluded high recruiting centres. All centres started as controls and were followed by 60 days of observation. We used block randomisation. The primary outcome was a 20% increase of recruitment within 60 days post intervention compared within 60 days pre intervention. Secondary outcomes were comparing recruitment between different types of centres, that is small versus large or experienced versus non-experienced centres, and university versus non-university hospitals. In exploratory analyses, recruitment within 30 days post versus 30 days pre intervention was compared.

**Results:**

The recruitment increased by 10% at 60 days. We noticed a short-lived increase of 23% the first month. The increased recruitment was most pronounced in low-recruiting, small and non-university hospitals. The recruitment of patients increased after the first contact with the centres where we announced that there would be a conference.

**Conclusion:**

A teleconference with the study personnel and the head of department increased the recruitment by 23% within 30 days and by 10%, 60 days post intervention in this embedded RCT. This implies that this structured intervention aimed at increased recruitment was short-lived and would need frequent repetitions in order to be effective.

## Introduction

Randomised controlled trials (RCTs) are the gold standard for evaluation treatments. Nevertheless, recruitment into trials is still challenging and many trials fail to meet their recruitment goals within the predetermined time (1). Slow recruitment can prolong studies and may also increase costs, thus requiring extended funding (2). A prematurely closed study could also compromise the research question, leading to failure to answer important questions. In the UK, finding methods to enhance recruitment in RCTs has been identified as the highest priority (3). Possible barriers are lack of time, resources, or experience. Other contributing factors are competing trials and a study protocol that is difficult to implement in the daily routine at the clinic (4, 5). There is a clear need for evidence-based recruitment strategies (6–8).

Despite the fact that several trials have explored possibilities to enhance recruitment surprisingly few have proven to be generalisable (9). Among things that have been tested are closer contact with the coordination centre by telephone or emails, individual feedback on recruitment, re-visits to review the trial protocol or other educational packages, changes in the consent process, and financial incentives; all with marginal effects on recruitment (9).

Trialists are advised to include study recruitment strategies within their trials (9). One way of doing that is to embed recruitment trials within a host trial. An embedded recruitment trial is a RCT in which the intervention to increase recruitment is tested in an ongoing host RCT (10–12). It can provide data that could affect the ongoing trial but also be of importance in the design and conduct of future trials. We chose a stepped wedge cluster randomised design since we believed that all centres might benefit from the intervention, The aim was to investigate whether a structured teleconference with the study personnel and the head of department accompanied by a commitment contract can enhance recruitment in the Efficacy oF Fluoxetine – a randomisEd Controlled Trial in Stroke (EFFECTS)-trial 60 days post intervention, compared to 60 days pre-intervention. A secondary aim was to elucidate the effect at 30-day post versus 30 days pre-intervention and to explore contributing factors to the difference in recruitment.

## Material and methods

This trial was an embedded study within EFFECTS, a RCT of fluoxetine for stroke recovery (12). Briefly, EFFECTS included adult individuals 2–15 days after stroke from 35 centres in Sweden (28 acute stroke units, 5 rehabilitation centres, and 2 geriatric rehabilitation centres). The primary objective of EFFECTS was to evaluate whether 20 mg fluoxetine would improve the functional outcome at 6 months. The original recruitment goal was that each centre should randomise at least two patients per month, but as the study progressed, we discovered a huge discrepancy between centres; 7 out of 35 centres recruited half of the patients.

**Figure 1:**
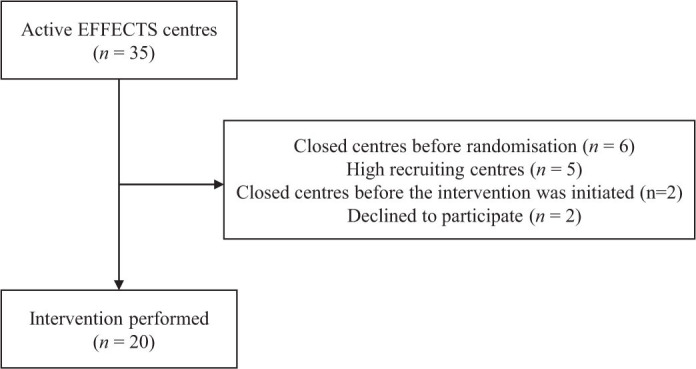
CONSORT flow ERUTECC.

When Enhancing Recruitment Using Teleconference and Commitment Contract (ERUTECC) started 9th September 2017, we had closed 6 of 35 centres in EFFECTS for administrative reasons. Hence, there were 29 active centres at the start of the intervention (Figure 1). We excluded the five high-recruiters (centres that recruited ≥ 2 patients/months) from participation in ERUTECC since we believed that they had reached their full potential and the intervention would be too weak for them. Two centres were closed between randomisation and the intervention (administrative reasons), and two centres declined to participate, leaving 20 centres for the intervention (Figure 1).

We chose the stepped wedge cluster design (13, 14), since we would be unable to conduct the intervention simultaneously at all centres. In addition, we have noticed a seasonal variation with significant lower recruitment during summer (June to August) and Christmas and New Year holidays. By using this design, we anticipated to reduce the seasonal variation. In a stepped wedge design centres receive the intervention, although the order is random. Moreover, we thought that all centres (except high recruiting) would benefit from this intervention.

We categorised the centres into three categories according to their average recruiting/month during an 18-month observation period between 1 March 2016 and 30 August 2017; low (< 0.5 patients/month), medium (between 0.5 and 2.0 patients/month), and high recruiter (> 2 patients/month). The rational for excluding the high recruiters was that we believed that the intervention would be futile. The reason for dividing centres into low- and medium recruiting was that we did not want to risk that all medium recruiting centres should fall into the same step, that is summer period, which so far has been a period of lower recruitment in the EFFECTS trial (data not shown). Another motive was that we suspected that the intervention might have different effects on low recruiters compared to medium centres. High, medium, and low recruitment rates were observed in both large university hospitals and smaller hospitals treating fewer stroke patients. Details of the centre are available in Supplement 1.

**Figure 2:**
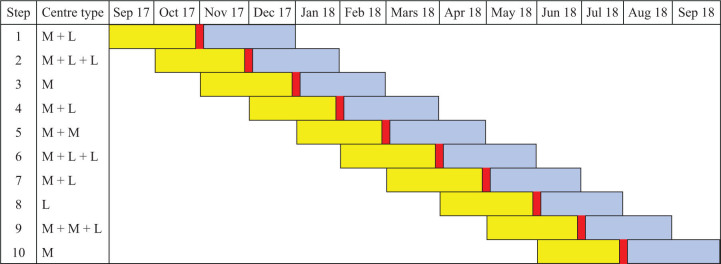
The ERUTECC stepped wedge trial design. M = medium recruiting centre and L = low recruiting centre. The yellow colour is 60 days before the intervention for each step, the blue colour is 60 days after the observation, and the red vertical bar indicates the time of the intervention.

The statistician was blinded and used stratified block randomisation to allocate the 20 centres into 10 clusters of two or three centres, leading to (at least) one medium and one low recruiting centre in each step. Because two centres were closed between randomisation and the intervention, and one centre was closed during the telephone conference, two clusters finally consisted of one centre. The time at which the centres started the intervention was randomly allocated. Every cluster consisted of 1–3 centres as shown in Figure 2.

Each centre was invited by email to the conference 1 month in advance (median 35 days). One week before the meeting an email with the attached agenda and a PowerPoint-presentation was sent to all participants (see Additional file 1). The meeting comprised a presentation of EFFECTS; background, rationale, aim, and update of recruitment. The discussion was related to barriers to recruitment at the local site with study personnel and the head of department and what could be done to increase recruitment. Full details of the intervention can be found in the published ERUTECC protocol (15). The Chief Investigator (EL) and the Trial Manager (EI) conducted the teleconference with the principal investigator and the trial team at each centre as well as the head of department.

The centres estimated how many patients they assumed they could randomise in the future and formulated a commitment contract regarding goals for recruitment that was duly signed. Our hypothesis was that a commitment contract would make the personnel put more effort into the intervention.

Before starting the intervention, we had a run-in period during which we measured how many patients each centre recruited over a 60-day period. We chose 60 days because many centres recruited small numbers of patients, 0–1 patients/months, and we estimated that a shorter period would lead to overly small numbers, and random variations.

### Outcomes

The primary outcome was the recruitment rate in the EFFECTS-trial 60 days post the ERUTECC intervention, compared with 60 days pre-intervention. Secondary outcomes were to compare the effect of the intervention on recruitment rates in:

1. Low- versus medium recruiting centres (according to their average recruiting/month in an 18-month observation period between 1 March 2016 and 30 August 2017)
2. Small versus large (≥ 500 stroke/year) stroke units
3. University hospitals versus non-university hospitals
4. Experienced centres versus non-Experienced centres. (An experienced centre was defined as a centre where both the investigator and the study nurse had been involved in five or more trials or had carried out their own research).

In exploratory analyses, we compared the recruitment 30 days before with 30 days after the intervention (post-hoc analysis).

The participants were not informed about the aim of ERUTECC or that the timing of the intervention was randomised or that we measured numbers of randomised patients before and after intervention, but they were fully aware of the fact that we wanted to enhance recruitment. The exact numbers of recruitment per centre were available at the public domain through a link that was updated in real time since the start of the EFFECTS study.

### Statistical analyses

For the primary outcome, we compared the numbers of included subjects 60 days before intervention with the numbers of subject 60 days post intervention. The null hypothesis was that there is no difference before and after. We considered a 20% increased recruitment rate as being a clear positive meaningful outcome. For the secondary outcomes, we compared the difference between the recruitment rates before and after intervention for different subgroups in the same way. We also tested for statistical significance between the subgroups with a generalised linear mixed model using the glmerMod function from lme4 library in the statistical programming language R.

## Results

We had one telephone conference with each centre and a total of 1–2 conferences were held every month between 9th September 2017 and 30th August 2018 and ended when all available centres had their intervention. The intervention did not improve the recruitment rate of 20% or more in EFFECTS. The inclusion rate before the intervention was 1.9 patients/centre/60 days and the inclusion rate after the intervention was 2.1 patients/centre/60 days, which is an increase in recruitment of 10% (Table 1).

**Table 1:** Primary and secondary outcome within 60 days before and after the intervention.

Primary and secondary outcomes measured within 60 days	Before	After	Difference	Improved patient recruitment of at least 20%	*P*
All centres (*n* = 20) patients/60 days	39	43	4 (10%)	no	
**Secondary outcomes measured within 60 days**					
Low recruiting centres (*n* = 9) patients/60 days	9	16	7 (78%)	yes	0.17
Medium recruiting centres (*n* = 11) patients/60 days	30	27	–3 (–10%)	no	
Small stroke units (*n* = 13) patients/60 days	25	31	6 (24%)	yes	0.42
Large stroke units (*n* = 4) patients/60 days	9	9	0 (0%)	no	
University hospitals (*n* = 4) patients/60 days	9	7	–2 (–22%)	no	0.46
Non-university hospitals including rehabilitation units (*n* = 16) patients/30 days	30	36	6 (20%)	yes	
Experienced centres (*n* = 9)	26	23	–3	no	0.67
Non-Experienced centres (*n* = 11)	13	20	7	no	

A ≥ 20% increase was defined as a positive outcome.

None of the statistical comparisons between the subgroups gave a significant result. However, the inclusion of patients increased by 150, 46, and 46% among the low recruiting, small, and non- university centres respectively the first month after the intervention (Table 2).

**Table 2:** Explorative outcomes within 30 days before and 30 days after the intervention.

Outcomes within 30 days	Before	After	Difference	Improved patient recruitment of at least 20%	*P*
All centres (*n* = 20) patients/30 days	22	27	5 (23%)	yes	
Low recruiting centres (*n* = 9) patients/30 days	4	10	6 (150%)	yes	0.12
Medium recruiting centres (*n* = 11) patients/30 days	18	17	–1 (–6%)	no	
Small stroke units (*n* = 13) patients/30 days	13	19	6 (46%)	yes	0.29
Large stroke units (*n* = 4) patients/30 days	7	6	1 (–14%)	no	
University hospitals (*n* = 4) patients/30 days	7	6	–1 (–14%)	no	0.32
Non-university hospitals (*n* = 16) patients/30 days	13	19	6 (46%)	yes	
Experienced centres (*n* = 9)	17	15	–2	No	0.72
Non-Experienced centres (*n* = 11)	5	12	7	No	

A ≥ 20% increase was defined as a positive outcome.

In a post-hoc analyse we found that recruitment increased within 30 days, especially among low recruiting centres, but also amongst small stroke units and non-university hospitals (Table 2).

In addition, we noticed that the inclusion rate increased after the first contact with each centre announcing a future telephone conference (Supplement 1).

During the teleconference, several things were highlighted. Study personnel at all centres considered EFFECTS’s research question to be important.

After the conference some centres changed their ways of working: the screening of patients was performed by the nurses instead of the physicians, other tasks related to the study (different scales, randomisation process) were also transferred to an experienced nurse instead of a physician, and most centres decided to make the study more visible by displaying a poster on the ward and information meetings for others working at the hospital.

## Discussion

Although a teleconference with the study personnel and the head of department accompanied by a commitment contract did not enhance recruitment by 20% or more 60 days post intervention compared to 60 days pre-intervention, recruitment increased among the low recruiting, small, and non-university hospitals. One possible reason why we saw this increase among the low recruiting hospitals was that they changed their organisation. For example, a low recruiting centre started by organised screening done by research nurses three times a week.

An important alteration for several centres was to increase the number of people working with the study and assign more tasks related to the study to an experienced nurse. It seems important to have a dedicated and organised research team with daily routines for doing research (16, 17).

It appears in our study that enthusiasm for a trial fades quickly. The effect of the intervention was short-lived (30 days) and did not last over a 60-day period. Regular contact from those leading the trial is of importance, this has also been shown by Fletcher et al. (7).

Another factor that the centres that increased recruitment included was making the trial visible at the hospital by setting up posters and regularly reminding of and talking about the trial at meetings, on rounds and with patients and relatives.

Our results showed that a phone call to the local centre, announcing a future conference, increased recruitment. This is in line with previous studies showing that regular contact with the study team increases recruitment (5, 18, 19). Our results suggest that it is important to show that those leading the trial care about the trial and the person with whom they are communicating, in order to maintain the commitment and to move the clinical trial forward.

Recruitment varied both between and within hospital types. Importantly, national registry data indicate that adjusted patient outcomes are comparable across university and non-university hospitals in Sweden (20) suggesting that organisational factors and volume, rather than hospital category, are the main drivers of differences.

A strength of our embedded recruitment trial is the stepped wedge randomised design. The design allowed us to carry out the intervention at all centres during the study period. We believed that all centres could benefit from the intervention, and in a stepped wedge design all centres are exposed. We were able to avoid the seasonal variation around Christmas, easter and especially in the summer. Moreover, it would have been impossible to do the intervention at all centres at the same time.

Our study has several limitations that may have influenced the results. Firstly, since the start for the EFFECTS-trial we had regular contact with most of the centres, working hard to encourage study personnel to find and include patients. We had consistently tried to identify barriers and find ways to enhance recruitment and there is a possibility that we had reached a ceiling effect in this regard and the intervention might have been too weak to achieve clear positive results. The idea was that the contract would be perceived as morally binding. In retrospect, more time designing and explaining what we meant by the contract could have been of importance. During our meetings with the various centres, we noticed that contract writing was viewed very differently. Some centres perceived it as encouraging, others were more reluctant to mention a recruitment figure per month.

Ultimately, we have tried to change the behaviour pattern for over 60 persons, and behaviour change is one of the hardest things to accomplish.

Secondly, we might be criticised for not including the top recruiting centres in ERUTECC. However, we believe that high recruiting centres already had reached their maximum recruitment potential and that the intervention would only have a minor effect. The fact that medium recruiting centres did not improve their inclusion strengthened that assumption. It was not a specific type of hospital that recruited many patients. Among the hospitals with high and medium recruitment were university hospitals as well as smaller hospitals with fewer stroke patients. A similarity of these sites was the presence of a committed investigator and nurse, along with well-developed routines for study implementation and patient identification.

Thirdly, the intervention might evolve during the study. For example, the agenda or the content of the teleconference might change during the study period. Participants in the host trial (EFFECTS) might get to know about the intervention and change their behaviour before the planned intervention.

Finally, two centres declined to be a part of the intervention, and this could have had an impact on the results. One of them increased their recruitment rate after being invited to participate in the intervention and that is not in our results. Two centres were closed for administrative reasons before the intervention was initiated. The local PI lacked motivation and for a long time had failed to include any patients. If we had managed to enthuse them, maybe the outcome would have been different.

## Conclusion

A teleconference with the study personnel and the head of department together with commitment contract did not increase recruitment to more than 20% in a RCT 60 days post intervention. However, recruitment did increase the first month, especially at low-recruitment centres.

## Supplementary Material





## Data Availability

The dataset for this study will be made available by the corresponding authors on reasonable request.

## Trial registration

The Northern Ireland Hub for Trials Methodology Research Studies Within a Trial repository (SWAT 58) on 30 April 2017 (20).

EFFECTS was registered in ClinicalTrials.gov, ID: NCT02683213.
