# Supplementary material for: Enhancing Recruitment Using Teleconference and Commitment Contract (ERUTECC): a stepped wedge cluster randomised trial within the EFFECTS trial
Source: Ups J Med Sci. 2025 Dec 1;130:10.48101/ujms.v130.12897. doi: 10.48101/ujms.v130.12897 (PMC12671279; doi:10.48101/ujms.v130.12897)
Supplement: Supplementary file 1 [file UJMS-130-12897-s1.pdf]

Supplement 1.

This appendix contains supplemental material related to the manuscript "Enhancing Recruitment Using Teleconference and Commitment Contract (ERUTECC): a stepped wedge cluster randomised trial within the EFFECTS trial". The tables provide detailed information on centre classification and recruitment patterns. Explanatory notes are given as footnotes to each table. A complete list of centres in the EFFECTS trial is provided, with indication of participation in the ERUTECC intervention.

Table 1. Classification of centres in EFFECTS and participation in ERUTECC

Centre ID	Centre name	Type of centre	Number of stroke /year *	Stroke unit size †	Recruiting level §	Patients per month ¶	Experience #	Comment	Participation in ERUTECC ‡
**1**	Danderyd Hospital	Non-university hospital	829	Large	High	3.6	Experienced		No
**2**	Karolinska University Hospital Solna	University hospital	519	Large	High	2.9	Experienced		No
**3**	Skaraborg Hospital Skövde	Non-university hospital	394	Small	High	2.2	Experienced		No
**4**	Hässleholm hospital	Non-university hospital	189	Small	Medium	0.5	Experienced		Yes
**5**	Uppsala University Hospital	University hospital	510	Large	Medium	0.9	Experienced		No
**6**	Karolinska University Hospital Huddinge	University hospital	415	Small	Medium	0.5	Non-Experienced	Closed centre	No
**7**	Mora Hospital	Non-university hospital	227	Small	High	1.9	Experienced		No
**8**	Falu Hospital	Non-university hospital	446	Small	Low	0.2	Non-Experienced		Yes
**9**	Skaraborg Hospital Lidköping	Non-university hospital	169	Small	Low	0.3	Experienced	Closed centre	No
**10**	Capio St. Göran Hospital	Non-university hospital	637	Large	Medium	1.9	Experienced		Yes
**11**	Visby Hospital	Non-university hospital	121	Small	Low	0.2	Experienced	Closed centre	No
**12**	University Hospital of Umeå	University hospital	345	Small	Low	0.4	Experienced		Yes
**13**	Kristianstad Central Hospital	Non-university hospital	137	Small	Low	0.4	Non- Experienced		Yes
**14**	Norrtälje Hospital	Non-university hospital	137	Small	Low	0.1	Non-Experienced		Yes
**15**	Helsingborg Hospital	Non-university hospital	417	Small	Medium	0.5	Non-Experienced		Yes
**16**	Skåne University Hospital Malmö	University hospital	603	Large	Medium	0.8	Experienced		Yes
**17**	Halland Hospital Halmstad	Non-university hospital	395	Small	Medium	1.7	Experienced		Yes

Centre ID	Centre name	Type of centre	Number of stroke /year *	Stroke unit size †	Recruiting level §	Patients per month ¶	Experience #	Comment	Participation in ERUTECC ‡
**18**	Mälarsjukhuset Eskilstuna	Non-university hospital	300	Small	Low	0.4	Non-Experienced		Yes
**19**	Rehab Station Stockholm	Rehabilitation clinic	NA	NA	Low	0.1	Non-Experienced		Yes
**20**	Skåne University Hospital Lund	University hospital	614	Large	Low	0.4	Experienced		Yes
**21**	Sundsvall Hospital	Non-university hospital	465	Small	High	3.3	Experienced		No
**22**	Sahlgrenska University Hospital	University hospital	674	Large	Medium	0.9	Experienced		Yes
**23**	Högsbo Rehabilitation Hospital	Rehabilitation clinic	NA	NA	Low	0.1	Non-Experienced	Closed centre	No
**24**	Stora Sköndal Neurologic Rehabilitation Clinic	Rehabilitation clinic	NA	NA	Low	0.7	Non-Experienced		Yes
**25**	Östersund Hospital	Non-university hospital	339	Small	Medium	1.2	Experienced		Yes
**26**	Alingsås Hospital	Non-university hospital	223	Small	Medium	1.9	Experienced		Yes
**27**	Ängelholm Hospital	Non-university hospital	230	Small	Medium	1.0	Non-Experienced		Yes
**28**	Stockholm Sjukhem Neurologic Rehabilitation Centre	Rehabilitation clinic	NA	NA	Medium	0.9	Non-Experienced		Yes
**29**	Örebro University Hospital	Non-university hospital	439	Small	Medium	0.6	Non-Experienced	Closed centre	No
**30**	Northern Älvsborg County Hospital Trollhättan	Non-university hospital	687	Large	Low	0.2	Non-Experienced	Closed centre	No
**31**	Stockholm Sjukhem Geriatrics	Rehabilitation clinic	NA	NA	Low	0.1	Non-Experienced	Closed centre	No
**32**	Västmanland Hospital Västerås	Non-university hospital	410	Small	Medium	0.6	Non-experienced		Yes
**33**	Dalen Hospital	Rehabilitation clinic	Not applicable	Not applicable	Low	0.1	Non-Experienced	Closed centre	No
**34**	Lindesberg Hospital	Non-university hospital	102	Small	Low	0.1	Non-Experienced	Declined	No
**35**	Hudiksvalls Hospital	Non-university hospital	140	Small	Low	0.4	Non-Experienced		Yes

NA denotes Not Applicable

* Reference to Riksstroke report 2018 (https://www.riksstroke.org/sve/forskning-statistik-och-verksamhetsutveckling/rapporter/arsrapporter/ Note the numbers of patient for 2017 are reported in the 2018 report.

† We defined a stroke unit as small if the number of stroke events was < 500 stroke/year, and large if the number of stroke events was ≥ 500 stroke/year.

§ We categorized the centres according to their average recruiting/month in an 18-month observation period between 1 March 2016 to 30 Aug 2017, into three levels: low (< 0.5 patients/month), medium (between 0.5-2.0 patients/month), and high recruiter (> 2 patients/month). This is 18 months before the ERUTECC started.

¶ Average inclusion of patient in the EFFECTS-trial, between March 2016 to Aug 2017.

An experienced centre was defined as one in which both the investigator and the study nurse had been involved in five or more clinical trials or had conducted their own research[1]

‡ A centre indicated as 'Yes' participated in the ERUTECC intervention.

Table 2. Recruitment in ERUTECC

Centre ID	Recruiting level *	Number of included patients between first contact and the intervention	Patients included 0-30 days before intervention	Patients included 31-60 days before intervention	Patients included 61-90 days before intervention	Patients included 0-30 days after intervention	Patients included 31-60 days after intervention	Patients included 61-90 days after intervention
**24**	Low	2	1	0	0	0	0	1
**35**	Low	1	0	1	1	3	2	1
**20**	Low	0	0	0	0	0	0	0
**14**	Low	0	0	1	0	0	0	1
**12**	Low	1	1	0	0	0	0	0
**13**	Low	0	0	0	1	2	0	0
**18**	Low	1	1	0	0	5	4	2
**8**	Low	0	0	0	0	0	0	0
**19**	Low	1	1	3	2	0	0	0
**26**	Medium	1	0	2	3	5	0	4
**25**	Medium	4	4	3	0	1	0	1
**4**	Medium	4	3	1	1	1	1	0
**17**	Medium	4	2	1	2	2	4	2
**27**	Medium	0	0	1	2	0	0	1
**16**	Medium	3	3	1	1	3	1	2
**15**	Medium	1	1	1	0	0	0	0
**22**	Medium	3	3	1	3	3	0	2
**32**	Medium	0	1	1	1	0	1	2
**28**	Medium	0	0	0	0	2	1	2
**10**	Medium	1	1	0	1	0	2	2

Recruitment numbers are given as absolute patient counts for consecutive 30-day periods before and after the intervention.
* We categorized the centres according to their average recruiting/month in an 18 months observation period between 1 March 2016 to 30 Aug 2017, into three levels: low (< 0.5 patients/month), medium (between 0.5-2.0 patients/month), and high recruiter (> 2 patients/month). This is 18 months before the ERUTECC started.

1. Isaksson E, Wester P, Laska AC, et al. Identifying important barriers to recruitment of patients in randomised clinical studies using a questionnaire for study personnel. *Trials* 2019; 20: 618. 2019/11/02. DOI: 10.1186/s13063-019-3737-1.
