# Supplementary material for: Enhancing Recruitment Using Teleconference and Commitment Contract (ERUTECC): a stepped wedge cluster randomised trial within the EFFECTS trial
Source: Ups J Med Sci. 2025 Dec 1;130:10.48101/ujms.v130.12897. doi: 10.48101/ujms.v130.12897 (PMC12671279; doi:10.48101/ujms.v130.12897)
Supplement: Supplementary file 2 [file UJMS-130-12897-s2.pptx]

## Slide 1
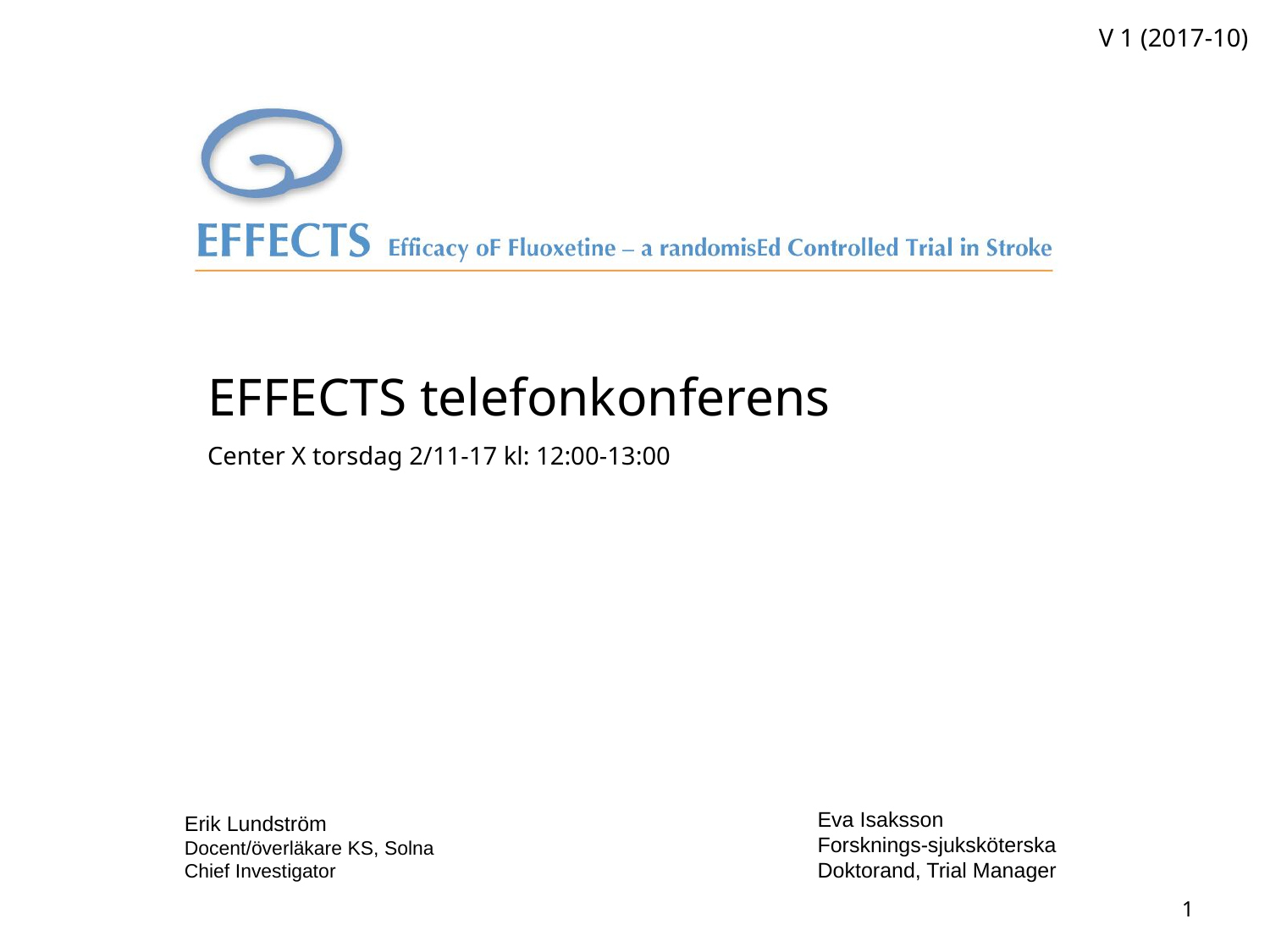

V 1 (2017-10)
EFFECTS telefonkonferens
Center X torsdag 2/11-17 kl: 12:00-13:00
Eva Isaksson
Forsknings-sjuksköterska
Doktorand, Trial Manager
Erik Lundström
Docent/överläkare KS, Solna
Chief Investigator
1

## Slide 2
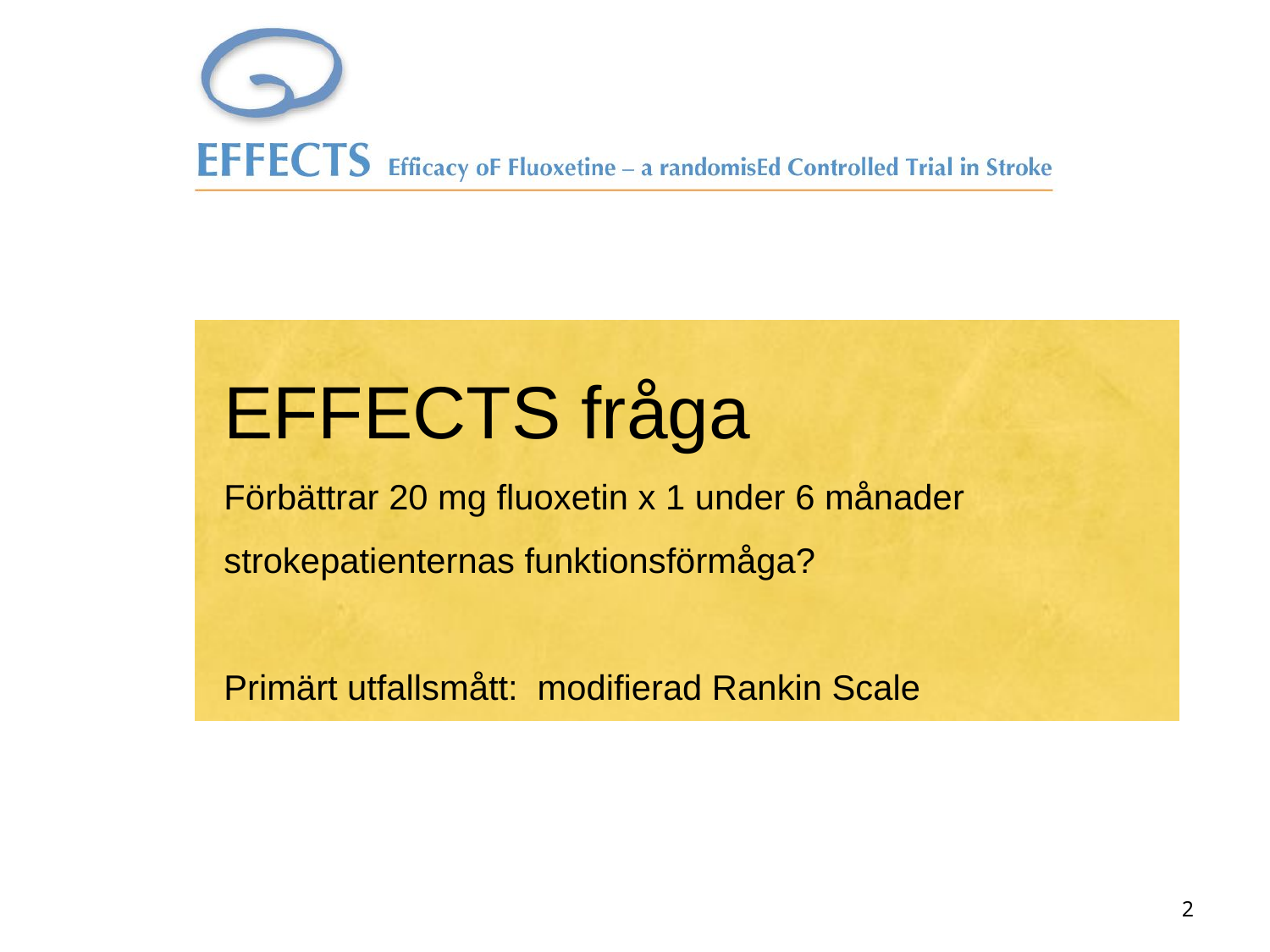

EFFECTS fråga
Förbättrar 20 mg fluoxetin x 1 under 6 månader
strokepatienternas funktionsförmåga?
Primärt utfallsmått: modifierad Rankin Scale
2

## Slide 3
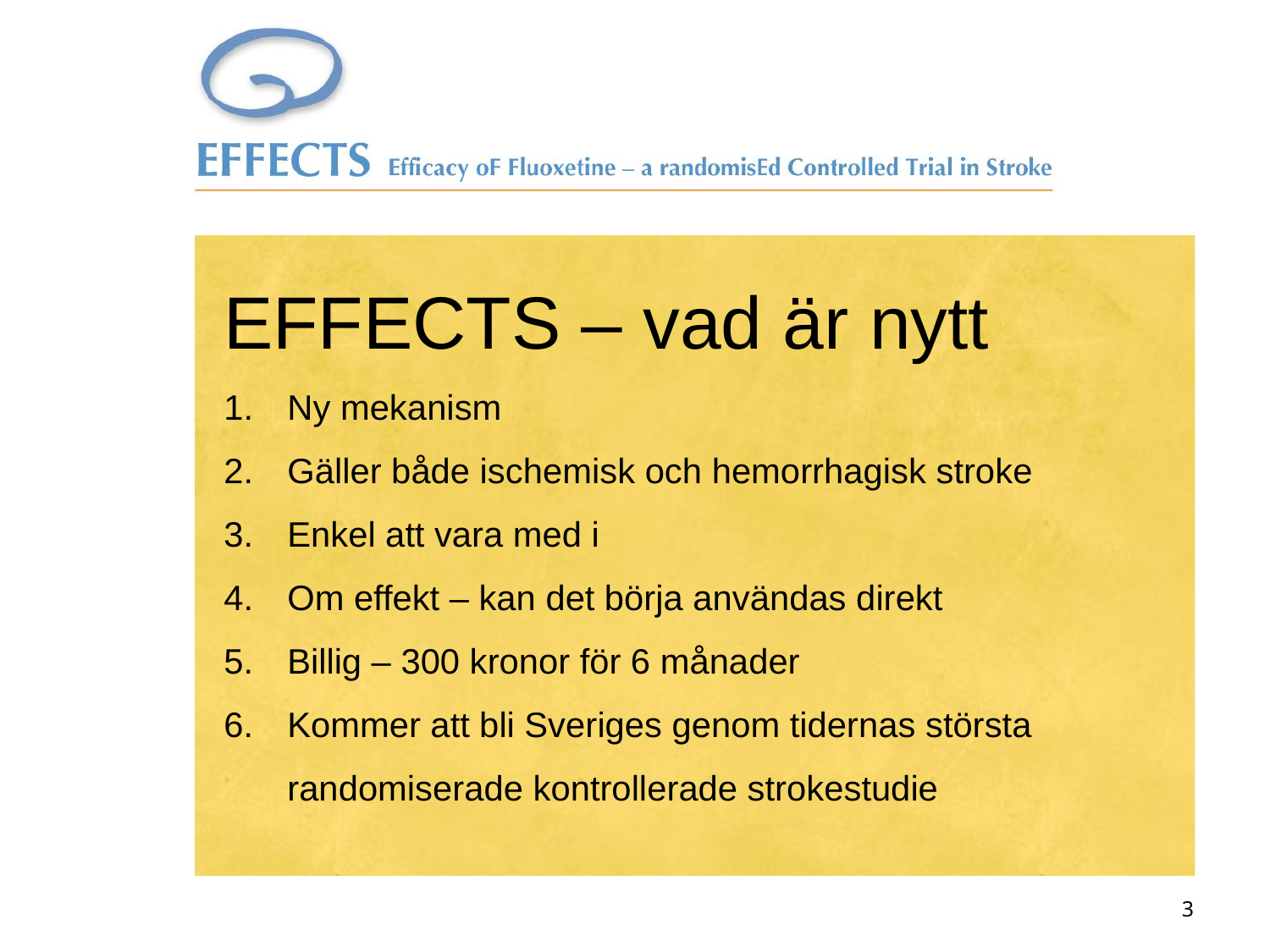

EFFECTS – vad är nytt
Ny mekanism
Gäller både ischemisk och hemorrhagisk stroke
Enkel att vara med i
Om effekt – kan det börja användas direkt
Billig – 300 kronor för 6 månader
Kommer att bli Sveriges genom tidernas största randomiserade kontrollerade strokestudie
3

## Slide 4
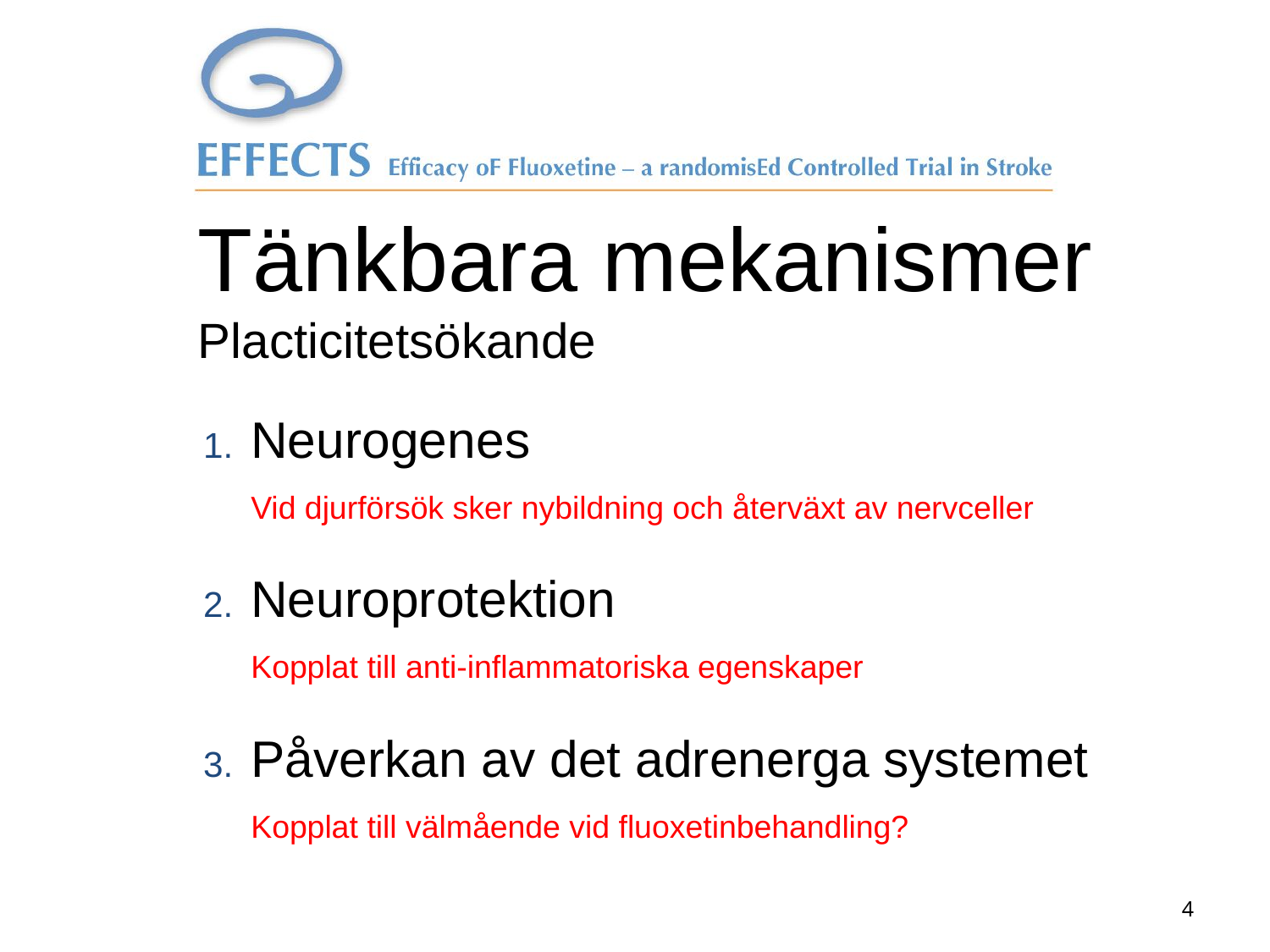

Tänkbara mekanismer
Placticitetsökande
Neurogenes
Vid djurförsök sker nybildning och återväxt av nervceller
Neuroprotektion
Kopplat till anti-inflammatoriska egenskaper
Påverkan av det adrenerga systemet
Kopplat till välmående vid fluoxetinbehandling?
4

## Slide 5
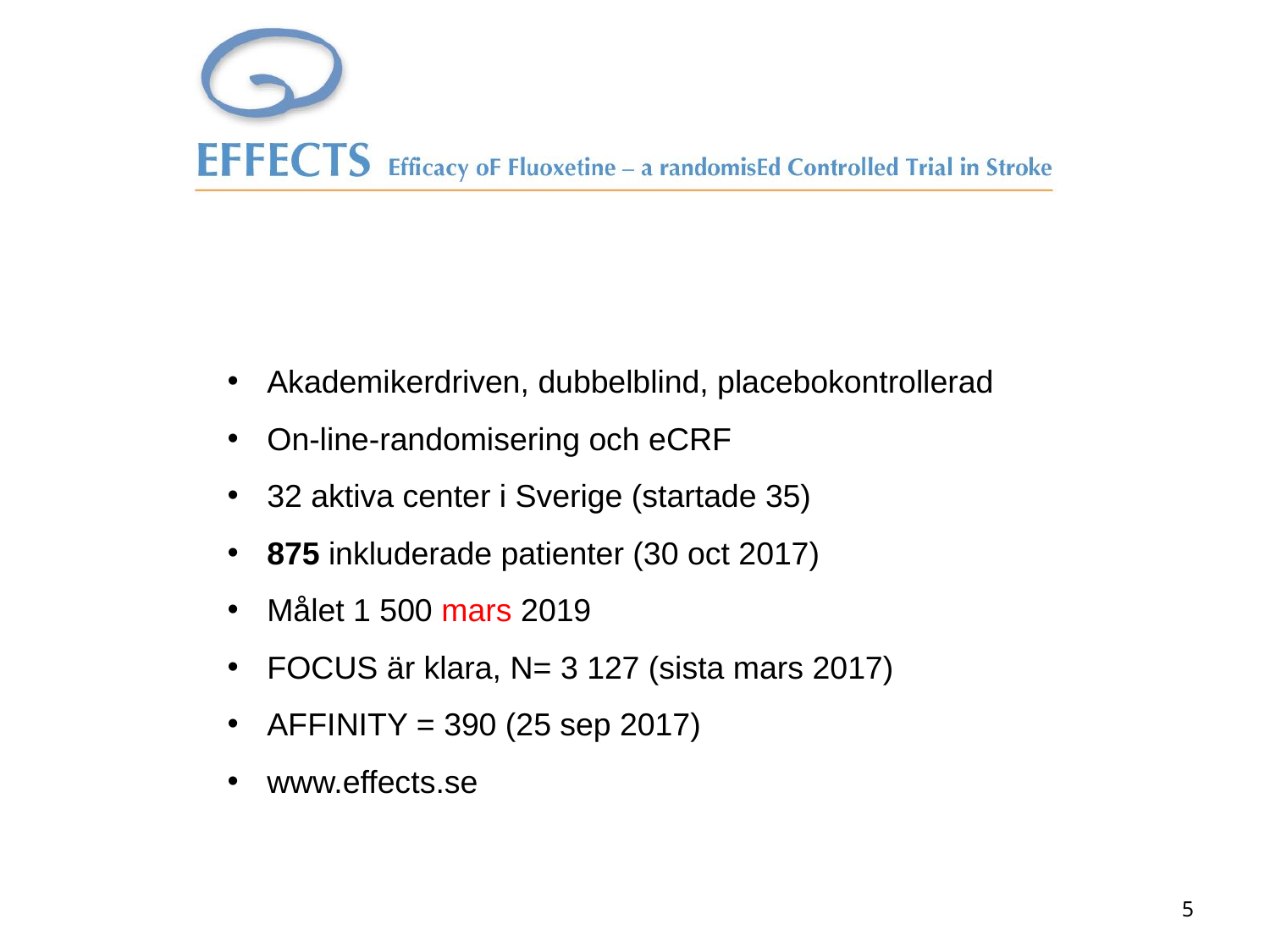

Akademikerdriven, dubbelblind, placebokontrollerad
On-line-randomisering och eCRF
32 aktiva center i Sverige (startade 35)
875 inkluderade patienter (30 oct 2017)
Målet 1 500 mars 2019
FOCUS är klara, N= 3 127 (sista mars 2017)
AFFINITY = 390 (25 sep 2017)
www.effects.se
5

## Slide 6
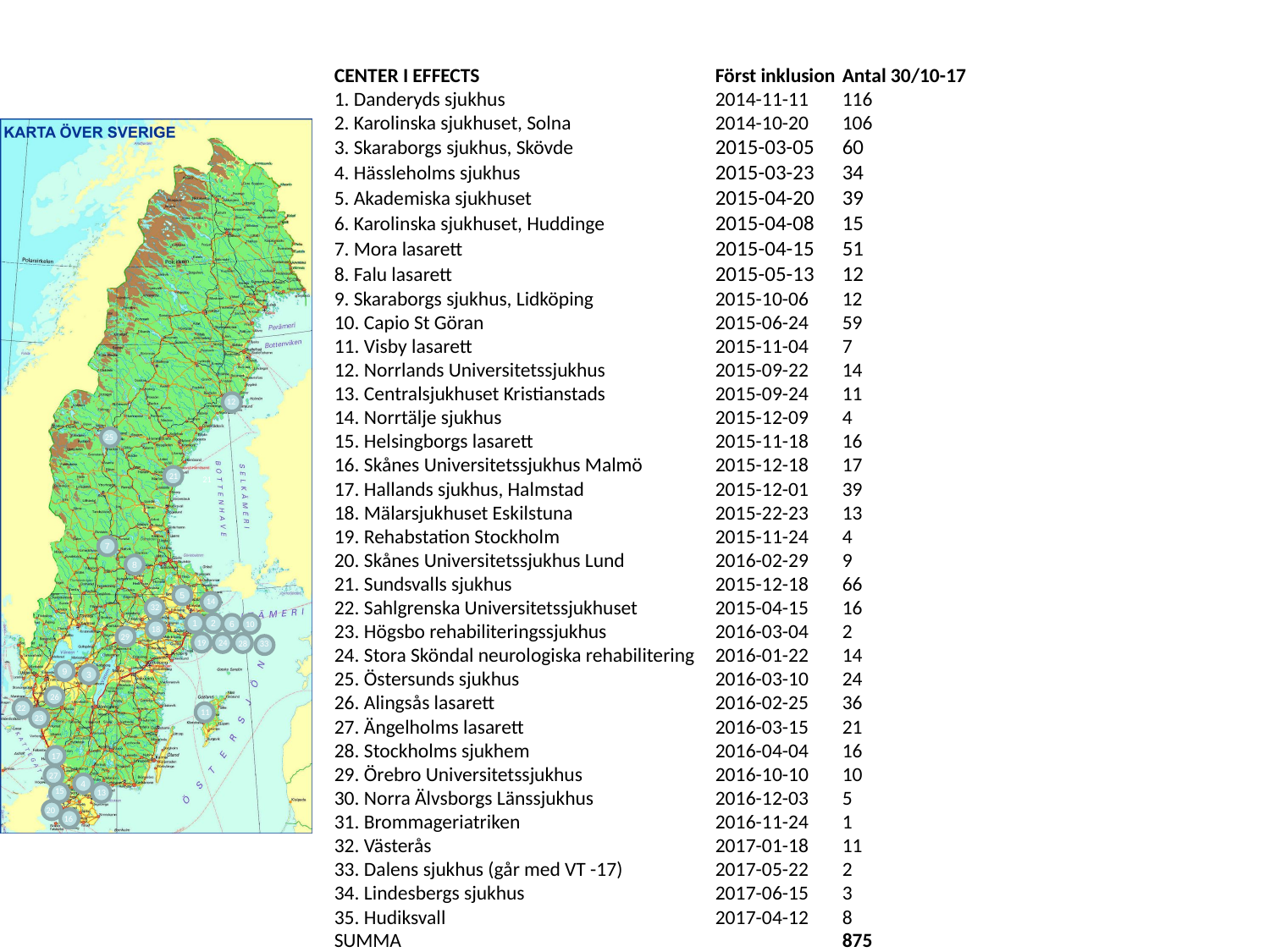

CENTER I EFFECTS		Först inklusion	Antal 30/10-17
1. Danderyds sjukhus		2014-11-11	116
2. Karolinska sjukhuset, Solna		2014-10-20	106
3. Skaraborgs sjukhus, Skövde		2015-03-05	60
4. Hässleholms sjukhus		2015-03-23	34
5. Akademiska sjukhuset		2015-04-20	39
6. Karolinska sjukhuset, Huddinge	2015-04-08	15
7. Mora lasarett		2015-04-15	51
8. Falu lasarett			2015-05-13	12
9. Skaraborgs sjukhus, Lidköping	2015-10-06	12
10. Capio St Göran		2015-06-24	59
11. Visby lasarett		2015-11-04	7
12. Norrlands Universitetssjukhus	2015-09-22	14
13. Centralsjukhuset Kristianstads	2015-09-24	11
14. Norrtälje sjukhus		2015-12-09	4
15. Helsingborgs lasarett		2015-11-18	16
16. Skånes Universitetssjukhus Malmö	2015-12-18	17
17. Hallands sjukhus, Halmstad		2015-12-01	39
18. Mälarsjukhuset Eskilstuna		2015-22-23	13
19. Rehabstation Stockholm		2015-11-24	4
20. Skånes Universitetssjukhus Lund	2016-02-29	9
21. Sundsvalls sjukhus		2015-12-18	66
22. Sahlgrenska Universitetssjukhuset	2015-04-15	16
23. Högsbo rehabiliteringssjukhus	2016-03-04	2
24. Stora Sköndal neurologiska rehabilitering	2016-01-22	14
25. Östersunds sjukhus		2016-03-10	24
26. Alingsås lasarett		2016-02-25	36
27. Ängelholms lasarett		2016-03-15	21
28. Stockholms sjukhem		2016-04-04	16
29. Örebro Universitetssjukhus		2016-10-10	10
30. Norra Älvsborgs Länssjukhus	2016-12-03	5
31. Brommageriatriken		2016-11-24	1
32. Västerås			2017-01-18	11
33. Dalens sjukhus (går med VT -17)	2017-05-22	2
34. Lindesbergs sjukhus		2017-06-15	3
35. Hudiksvall			2017-04-12	8
SUMMA				875
12
25
21
21
7
8
5
14
32
10
1
2
6
18
29
19
24
28
33
9
3
26
22
11
23
17
27
4
15
13
20
16

## Slide 7
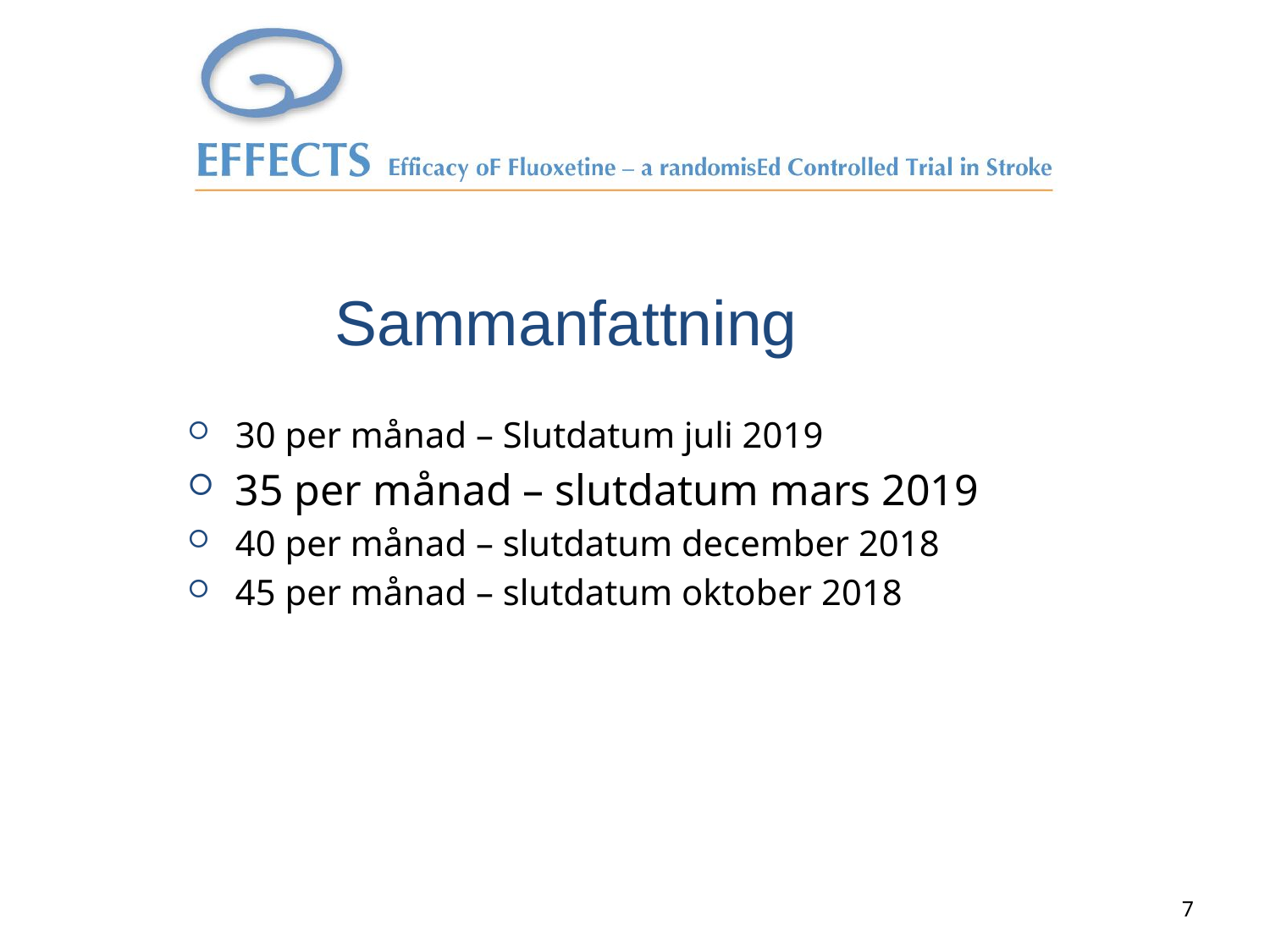

# Sammanfattning
30 per månad – Slutdatum juli 2019
35 per månad – slutdatum mars 2019
40 per månad – slutdatum december 2018
45 per månad – slutdatum oktober 2018
7

## Slide 8
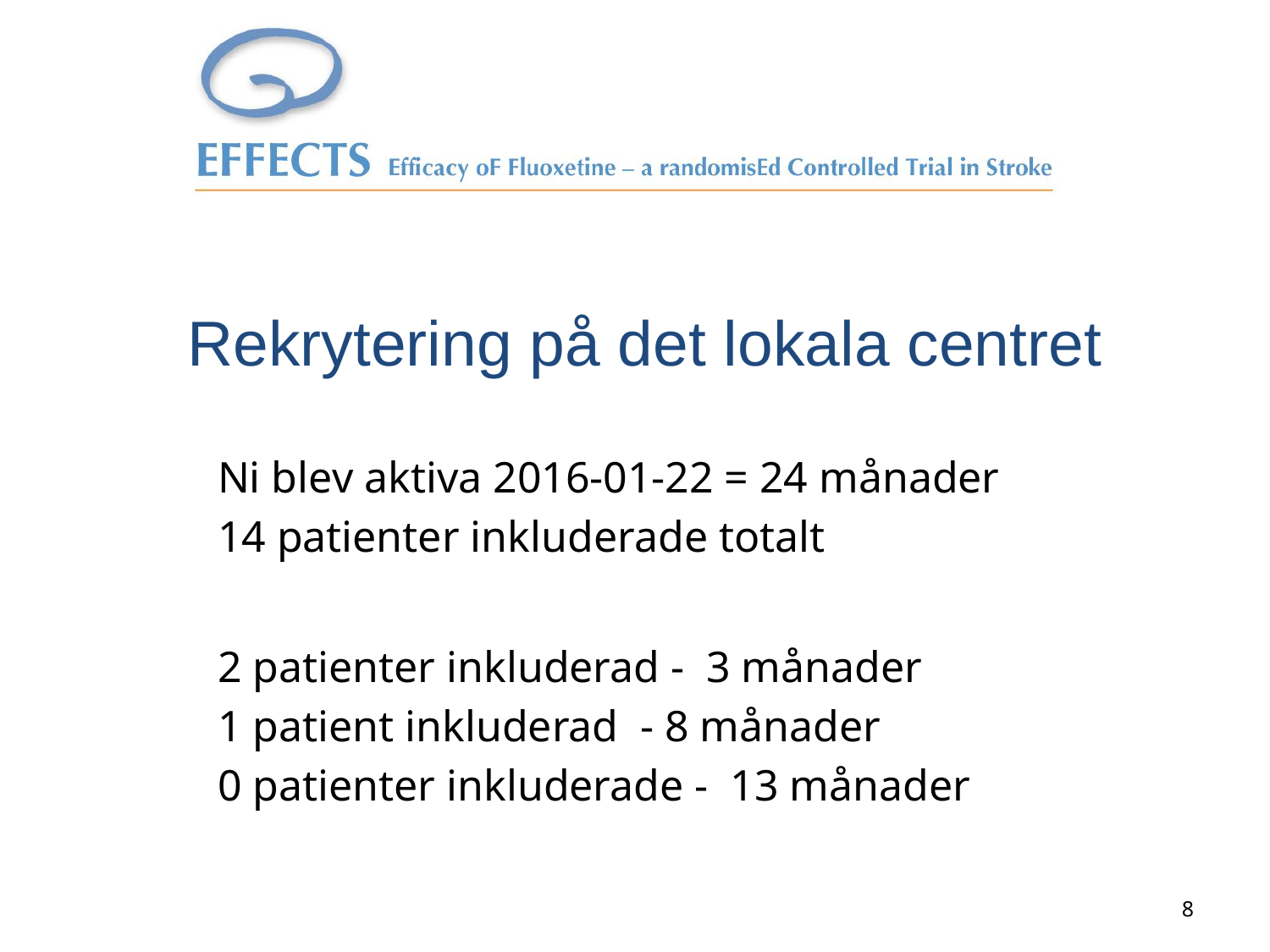

# Rekrytering på det lokala centret
Ni blev aktiva 2016-01-22 = 24 månader
14 patienter inkluderade totalt
2 patienter inkluderad - 3 månader
1 patient inkluderad - 8 månader
0 patienter inkluderade - 13 månader
8

## Slide 9
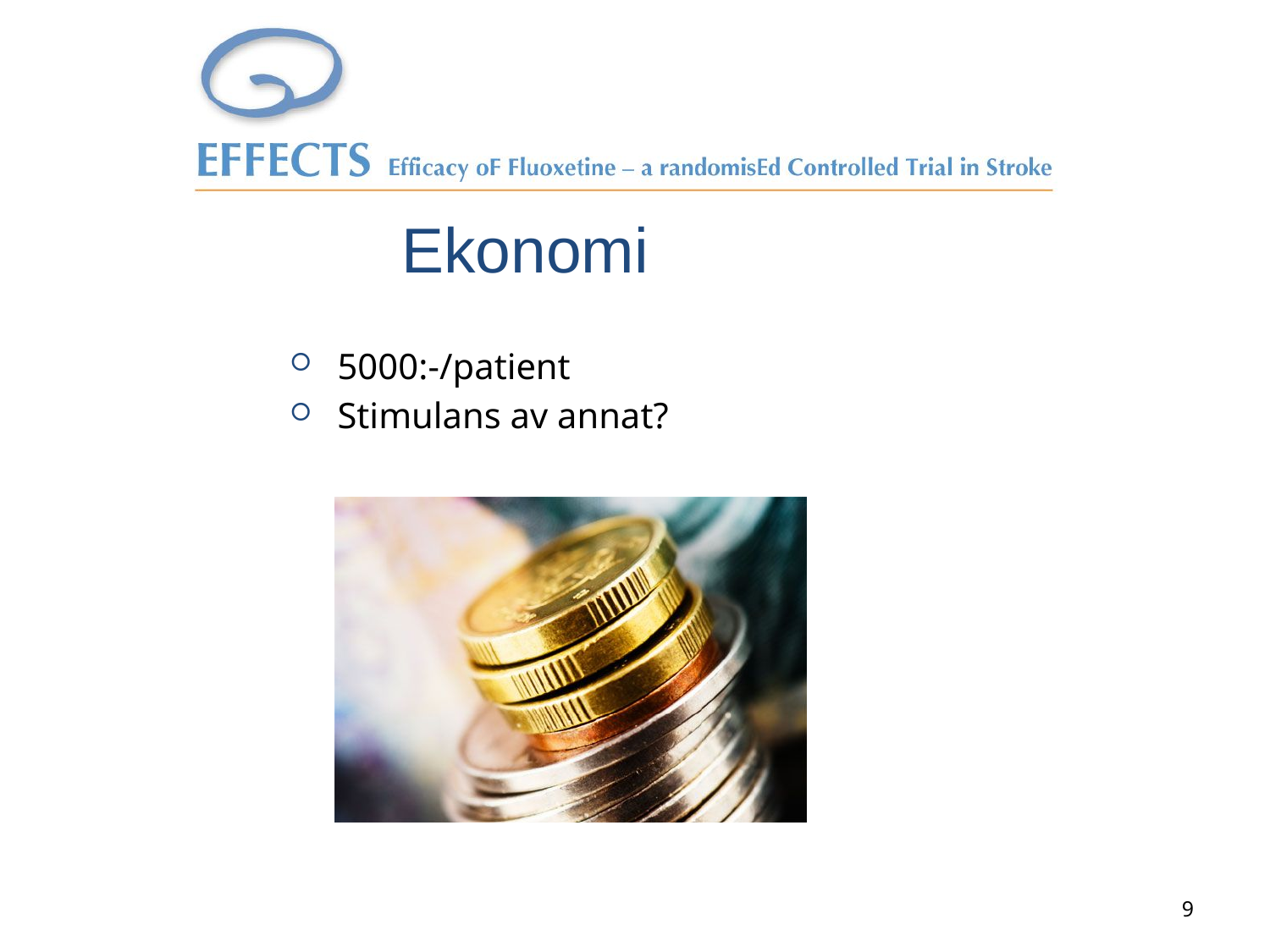

# Ekonomi
5000:-/patient
Stimulans av annat?
9

## Slide 10
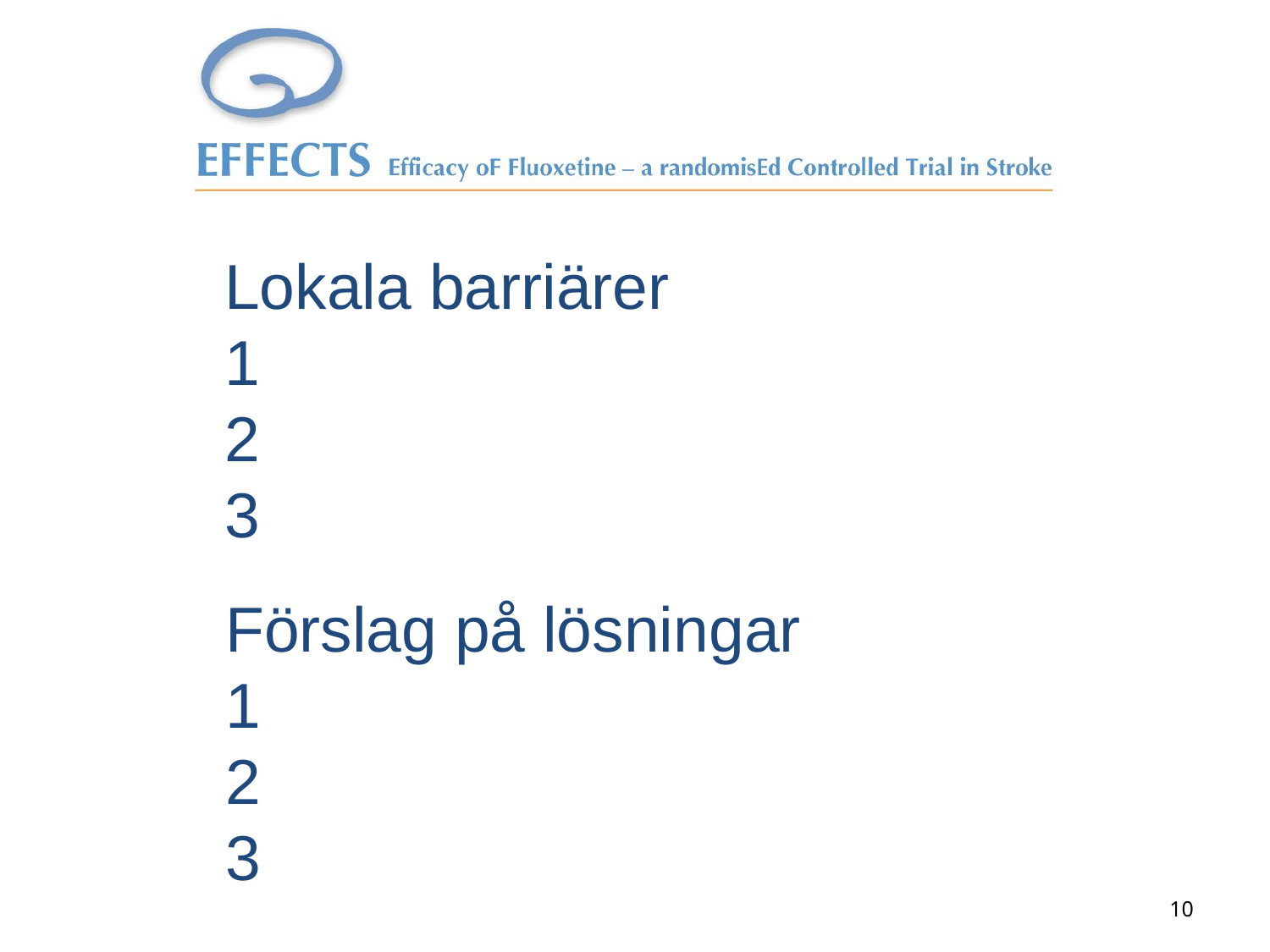

Lokala barriärer
1
2
3
# Förslag på lösningar 1 2 3
10

## Slide 11
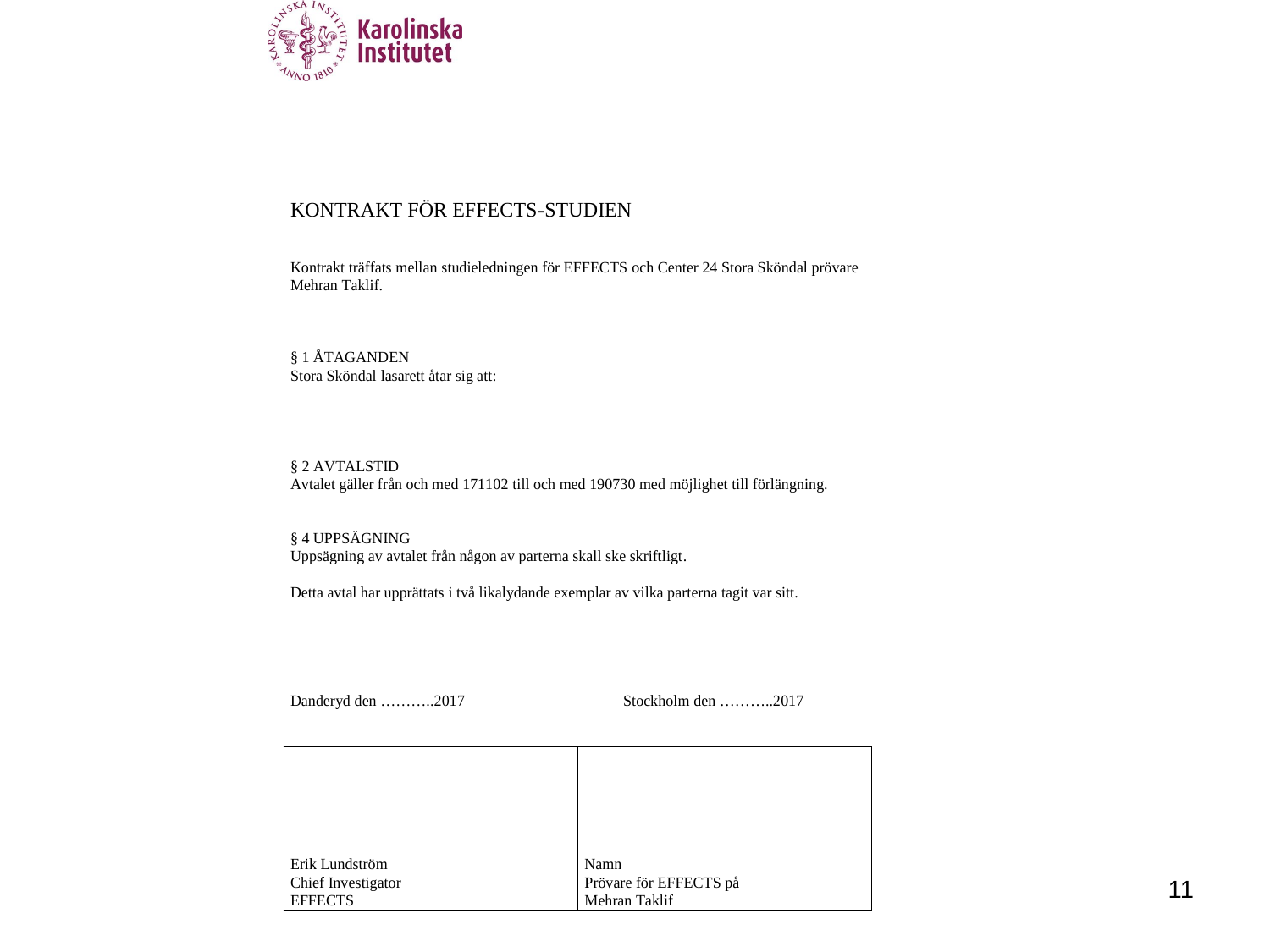

11

## Slide 12
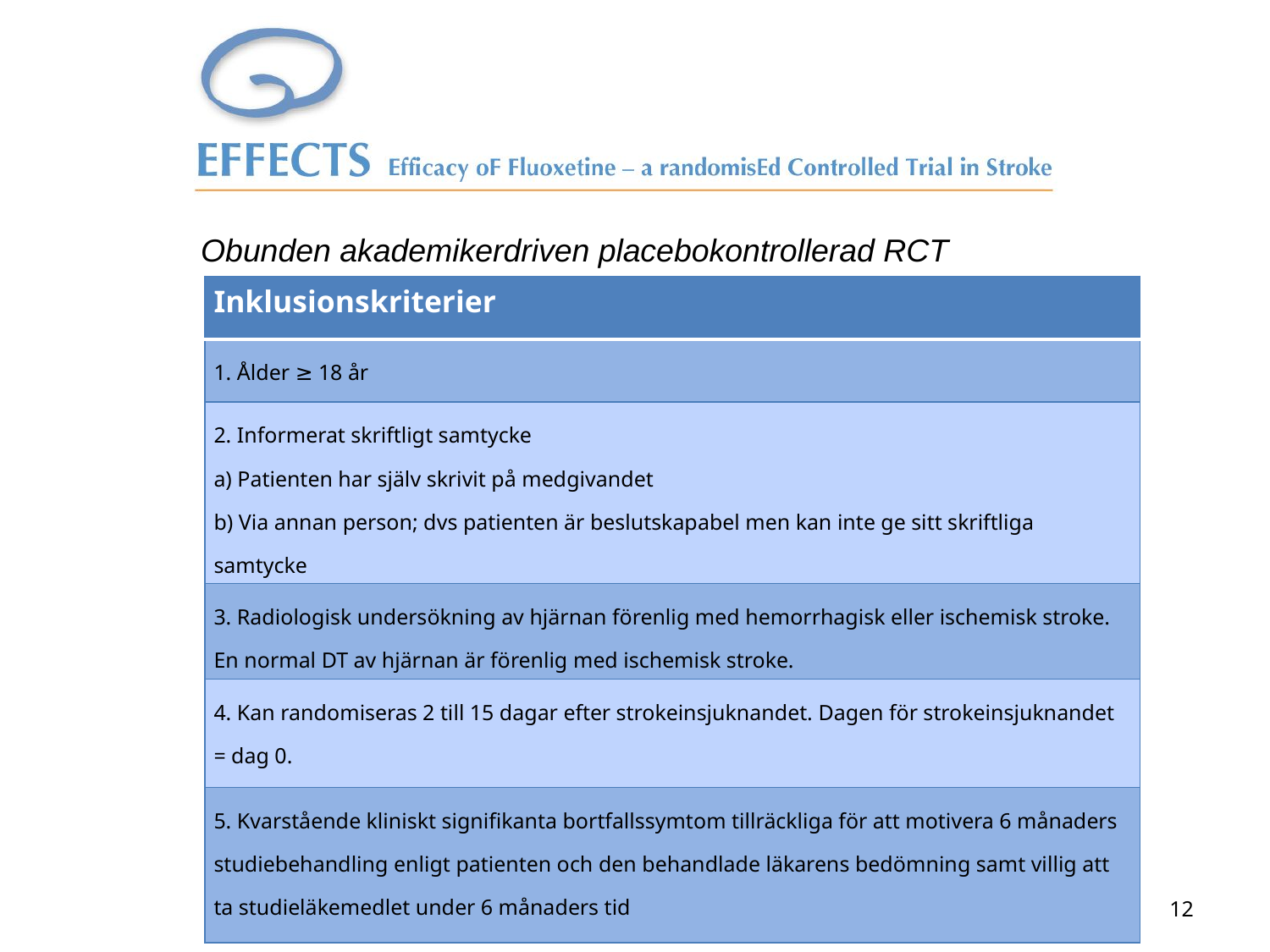

Obunden akademikerdriven placebokontrollerad RCT
| Inklusionskriterier |
| --- |
| 1. Ålder ≥ 18 år |
| 2. Informerat skriftligt samtycke a) Patienten har själv skrivit på medgivandet b) Via annan person; dvs patienten är beslutskapabel men kan inte ge sitt skriftliga samtycke |
| 3. Radiologisk undersökning av hjärnan förenlig med hemorrhagisk eller ischemisk stroke. En normal DT av hjärnan är förenlig med ischemisk stroke. |
| 4. Kan randomiseras 2 till 15 dagar efter strokeinsjuknandet. Dagen för strokeinsjuknandet = dag 0. |
| 5. Kvarstående kliniskt signifikanta bortfallssymtom tillräckliga för att motivera 6 månaders studiebehandling enligt patienten och den behandlade läkarens bedömning samt villig att ta studieläkemedlet under 6 månaders tid |
12

## Slide 13
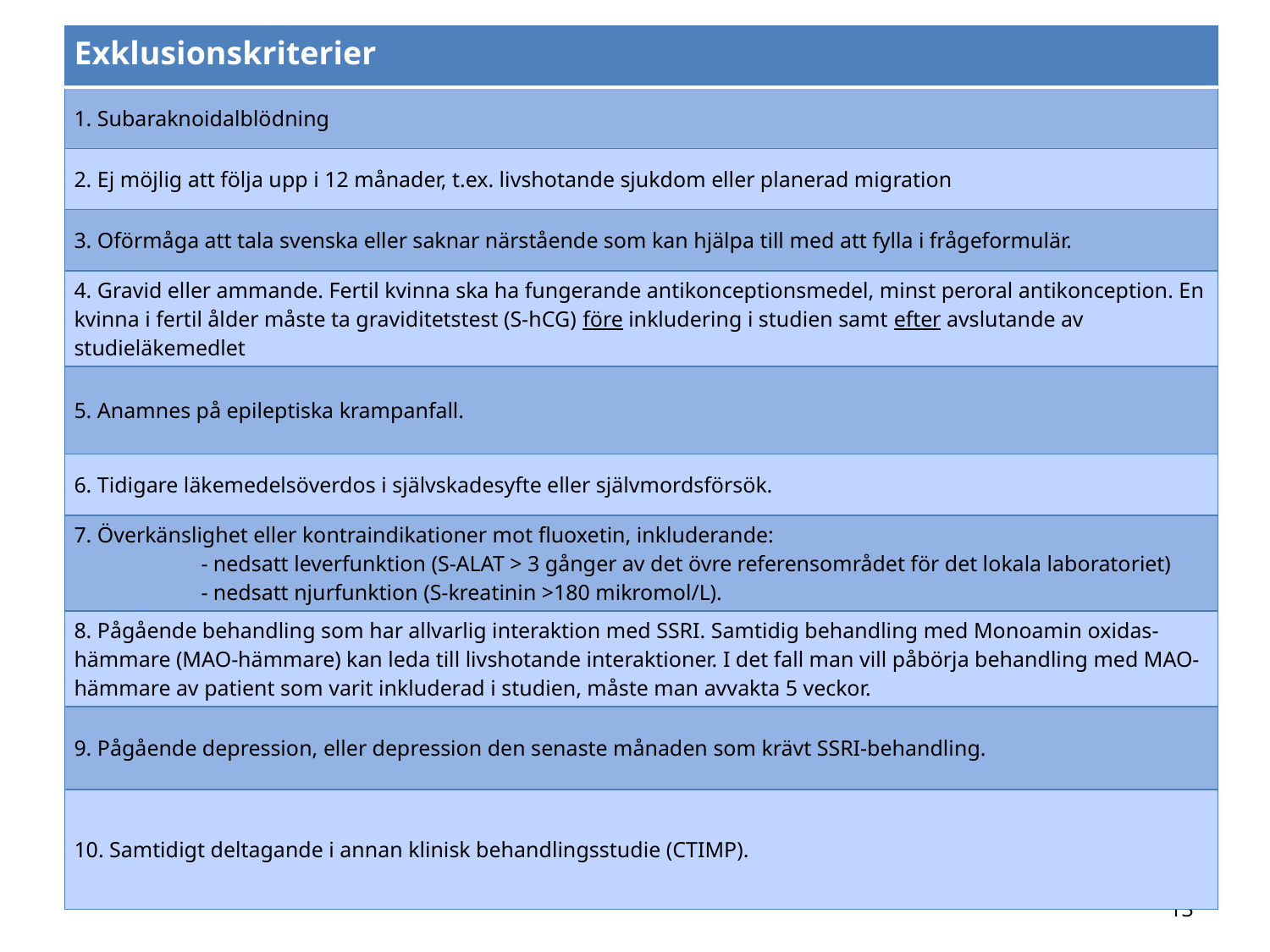

| Exklusionskriterier |
| --- |
| 1. Subaraknoidalblödning |
| 2. Ej möjlig att följa upp i 12 månader, t.ex. livshotande sjukdom eller planerad migration |
| 3. Oförmåga att tala svenska eller saknar närstående som kan hjälpa till med att fylla i frågeformulär. |
| 4. Gravid eller ammande. Fertil kvinna ska ha fungerande antikonceptionsmedel, minst peroral antikonception. En kvinna i fertil ålder måste ta graviditetstest (S-hCG) före inkludering i studien samt efter avslutande av studieläkemedlet |
| 5. Anamnes på epileptiska krampanfall. |
| 6. Tidigare läkemedelsöverdos i självskadesyfte eller självmordsförsök. |
| 7. Överkänslighet eller kontraindikationer mot fluoxetin, inkluderande: - nedsatt leverfunktion (S-ALAT > 3 gånger av det övre referensområdet för det lokala laboratoriet) - nedsatt njurfunktion (S-kreatinin >180 mikromol/L). |
| 8. Pågående behandling som har allvarlig interaktion med SSRI. Samtidig behandling med Monoamin oxidas-hämmare (MAO-hämmare) kan leda till livshotande interaktioner. I det fall man vill påbörja behandling med MAO-hämmare av patient som varit inkluderad i studien, måste man avvakta 5 veckor. |
| 9. Pågående depression, eller depression den senaste månaden som krävt SSRI-behandling. |
| 10. Samtidigt deltagande i annan klinisk behandlingsstudie (CTIMP). |
13

## Slide 14
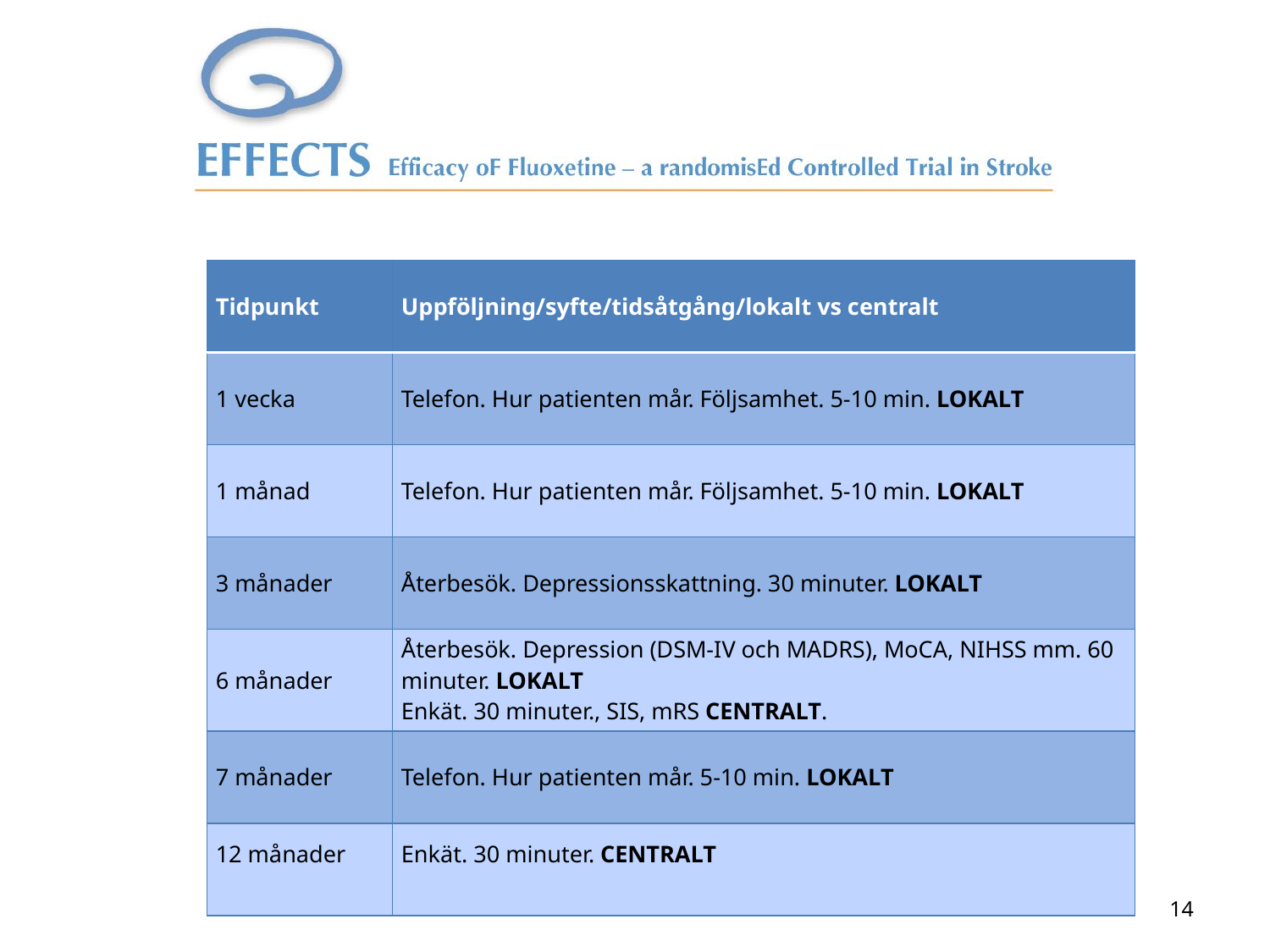

| Tidpunkt | Uppföljning/syfte/tidsåtgång/lokalt vs centralt |
| --- | --- |
| 1 vecka | Telefon. Hur patienten mår. Följsamhet. 5-10 min. LOKALT |
| 1 månad | Telefon. Hur patienten mår. Följsamhet. 5-10 min. LOKALT |
| 3 månader | Återbesök. Depressionsskattning. 30 minuter. LOKALT |
| 6 månader | Återbesök. Depression (DSM-IV och MADRS), MoCA, NIHSS mm. 60 minuter. LOKALT Enkät. 30 minuter., SIS, mRS CENTRALT. |
| 7 månader | Telefon. Hur patienten mår. 5-10 min. LOKALT |
| 12 månader | Enkät. 30 minuter. CENTRALT |
14
